# Transglutaminase 1: Emerging Functions beyond Skin

**DOI:** 10.3390/ijms251910306

**Published:** 2024-09-25

**Authors:** Sahar Ebrahimi Samani, Hideki Tatsukawa, Kiyotaka Hitomi, Mari T. Kaartinen

**Affiliations:** 1 Division of Experimental Medicine, Faculty of Medicine and Health Sciences, McGill University, Montreal, QC H3A 0C7, Canada; sahar.ebrahimisamani@mail.mcgill.ca; 2 Department of Basic Medicinal Sciences, Graduate School of Pharmaceutical Sciences, Nagoya University, Nagoya 464-8601, Japan; tatsukawa.hideki.i2@f.mail.nagoya-u.ac.jp (H.T.); hitomi@ps.nagoya-u.ac.jp (K.H.); 3 Faculty of Dental Medicine and Oral Health Sciences, McGill University, Montreal, QC H3A 1G1, Canada

**Keywords:** transglutaminase, transglutaminase 1, keratinocyte transglutaminase, epidermal transglutaminase

## Abstract

Transglutaminase enzymes catalyze Ca^2+^- and thiol-dependent posttranslational modifications of glutamine-residues that include esterification, hydrolysis and transamidation, which results in covalent protein–protein crosslinking. Among the eight transglutaminase family members in mammals, transglutaminase 1 (TG1) plays a crucial role in skin barrier formation via crosslinking and insolubilizing proteins in keratinocytes. Despite this established function in skin, novel functions have begun merging in normal tissue homeostasis as well as in pathologies. This review summarizes our current understanding of the structure, activation, expression and activity patterns of TG1 and discusses its putative novel role in other tissues, such as in vascular integrity, and in diseases, such as cancer and fibrosis.

## 1. Overview of Transglutaminases (TGs)

Transglutaminases (EC:2.3.2.13; Protein-glutamine γ-glutamyltransferases) are a family of crosslinking enzymes that catalyze Ca^2+^-ion and thiol-dependent posttranslational modifications of specific glutamine residues that, depending on the environment, can lead to transamidation (crosslinking), esterification, and hydrolysis of glutamines. In the mammalian TG family, there are eight TG isozymes, including TG1–7 and Factor XIII-A, which are distributed throughout the body but have often been assigned a name that reflects their most prevalent functions [1]; however, new findings over the decades have added to the repertoires of the enzymes in health and disease progression. In addition to being crucial for the formation of the skin barrier, blood clotting, and extracellular matrix stabilization, the TG enzymes play a role in the pathophysiology of several diseases, such as fibrosis, skin disorders and neurodegenerative conditions [2,3,4]. The best-known catalytic reaction of TGs is the transamidation reaction, which forms a covalent isopeptide bond (ε-γ-glutamyl)lysine bond) between glutamine and lysine residues. The isopeptide bond formation can induce protein oligomerization/polymerization and result in high molecular weight protein complexes and stabilize large protein networks, such as is seen in fibrin stabilization by Factor XIII-A. This covalent stabilization increases the resistance of proteins to proteolysis. TGs are also involved in polyamination of glutamines, which involves covalent crosslinking of primary amines such as spermine, spermidine, putrescine and proteins [5,6]. Similarly, TGs can link monoamines like dopamine and serotonin to glutamine residues [7]. When water is present, and no poly/monoamines or protein-bound lysine residues are available, TGs convert glutamine to glutamic acid. This reaction is the main pathophysiological mechanism behind celiac disease, where TG activity from TG2 deaminates gluten peptides, which creates T-cell epitopes that trigger an immune response against gluten. The first ever randomized clinical trial using a drug against a TG enzyme was to prevent gluten deamination with TG2 inhibitor, ZED1227 [8].

The TG catalysis process is defined by the glutamine-containing substrate, which interacts with the TG active site Cys to form a covalently linked glutamylthioester intermediate. This is followed by a second intermediate, acylenzyme formation and release of ammonia. The initial acylation step occurs upon the binding of (1) a second amine substrate (transamidation), (2) water (hydrolysis), or (3) an alcohol (esterification), resulting in the release of the product from the active site. Thus, TGs have less preference for the amine-donor substrate [1,9,10,11].

The presence of TG activity and crosslinks in mammalian epidermis was first suggested in the 1970s [12,13,14], with later studies confirming that these crosslinks are primarily located in the cornified envelope, where they contribute to the insolubility of the protein lattice in human epidermal keratinocytes [15]. TG1 was subsequently identified in 1985 from cultured human epidermal cells, where it was found to play a crucial role in forming a covalently crosslinked protein layer beneath the plasma membrane during the final stages of keratinocyte differentiation [16]. This finding was further supported by similar discoveries in specific fractions of epidermal keratinocytes [17,18,19] and the identification of numerous mutations in its gene in patients with congenital lamellar ichthyosis, which results in inactive enzymes and defective cornification of skin (more below) [20,21]. In mice, global deletion of the *Tgm1* gene is lethal within a few hours after birth due to a defective skin barrier that causes dehydration of the pups [22]. Beyond its ‘name and fame’ as skin TG, novel functions for TG1 are beginning to emerge in various physiological and pathological processes. This review aims to provide an overview of TG1’s structure, enzymatic activity, expression patterns, and possible novel roles in different tissues and disease contexts.

## 2. Structure of TG1

**Figure 1:**
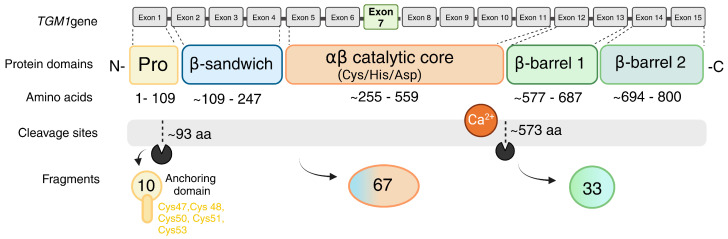
Scheme of human keratinocyte *TGM1*; gene, protein domains activating cleavage sites. *TGM1* gene is encoded by 15 exons which create conserved TG enzyme domains; β-sandwich, αβ-catalytic core, and b-barrels 1 and 2. Like with other TGs, amino acids Cys377, His436 and Asp459 form the catalytic triad in the catalytic core where Cys residue, residing in exon 7, plays the critical role. Ca^2+^ sites are proposed to reside within residues 499, 501, 548, 553. TG1 contains a pro-peptide which forms the anchoring domain containing the Cys cluster, which allows its insertion into the inner leaflet of the plasma membrane in keratinocytes [21,23,24,25,26,28]. Everything in the scheme is approximate. Created in BioRender. Kaartinen, M. (2024) BioRender.com/i20v413.

Transglutaminase 1 (TG1), also known as epidermal TG, keratinocyte TG, and protein-glutamine gamma-glutamyltransferase K (KTG, TGK, TGase K), is encoded by the *Tgm1* gene in mice, located on chromosome 14 C3 and comprising 18 exons. In humans, the *TGM1* gene spans 14,095 base pairs, including a 2454 bp coding sequence and consists of 15 exons, located on chromosome 14q11.2 [23,24,25,26]. The exons 3 to 14 are conserved between TG1 and other members of the TG family due to a shared evolutionary origin and functional similarities. All exon–intron junctional sequences adhere to the canonical GT-AG rule, ensuring proper splicing and accurate mRNA processing. The translation start site is located in the second exon, and the active site cysteine (Cys) residue of the enzyme is found in exon 7 [27] (Figure 1).

Based on the amino acid sequence, human TG1 is composed of five main domains: the anchoring domain (residues 1–92), the β-sandwich domain (residues 94–246), the catalytic core domain (residues 247–572), β-barrel 1 (residues 573–688) and β-barrel 2 (residues 689–817) [28]. Like other TGs, the active enzyme comprises four main domains, which for TG1 were also reported to be an N-terminal β-sandwich domain from residues Met-109 to Phe-247, encoded by exons 2, 3 and 4; an α/β catalytic core from Ile-255 to Pro-559 (exons 5–12); and two C-terminal β-barrel domains, domain 3 (exons 12–13) and domain 4 (exons 14–15), which include Glu-577 to Arg-687 and Leu-694 to Gly-800, respectively. In addition, TG1 has an additional N-terminal pro-peptide sequence, spanning from Met-1 to Met-109. The most important cleavage site is located at the end of this propeptide, leading to increased activity of TG1 compared to the full length of the enzyme [21]. The catalytic core contains the active site residues consisting of Cys377, His436 and Asp459, which forms the catalytic triad that is crucial for the transamidation activity of the enzyme. A conserved tryptophan residue in the catalytic core is also involved in the crosslinking/isopeptide bond formation via stabilization of the transition state during the formation of acylenzyme intermediate. TG1 is also unique in its function as a membrane-bound TG. The membrane insertion occurs via a cysteine cluster, i.e., five N-terminal cysteine residues (Cys47, Cys48, Cys50, Cys51, and Cys53) [29,30] which allow for TG1 lipid modification (palmitoylation) and membrane insertion (Figure 1).

## 3. Activation of TG1

**Figure 2:**
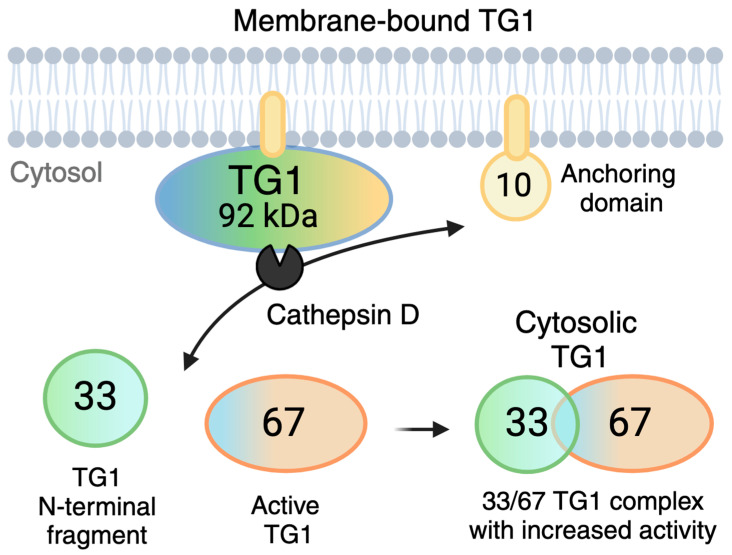
Activation of human keratinocyte TG1. TG1 is bound to the inner leaflet of the plasma membrane via an N-terminal anchoring domain that contains its pro-peptide region. TG1 can be cleaved by Cathepsin D, which creates a 67 kDa fragment that contains the enzyme active site, and a 33 kDa N-terminal fragment. The two fragments can form a complex in the cytosol to form a TG1 with further increased activity. Molecular weights are based on their running behavior in gels. Based on data from [29,30,32,34,35], it is not fully known if and how TG1 is activated in other tissues. Created in BioRender. Kaartinen, M. (2024) BioRender.com/g02n599.

TG1 and Factor XIII-A are two TG enzymes that have additional N-terminal pro-peptide sequences that can be proteolytically cleaved to enhance the enzyme activity [27]. It is important to note that the non-proteolyzed, full-length forms of these two enzymes can have TG activity (for example commercially available recombinant proteins), and activation by high Ca^2+^ and by substrate binding can also occur, as reported for FXIII-A [31]. The proteolytic activation of Factor XIII-A occurs at residues Arg37 and Gly38 in hemostasis via thrombin to induce rapid stabilization of fibrin clots [32,33]; however, TG1 activation appears to involve more complex processes, at least in keratinocytes. TG1 is anchored in a plasma membrane through a fatty acyl linkage at an N-terminal cysteine cluster that is located in the pro-peptide domain [29,30] (Figure 1 and Figure 2). *TGM1* encodes the transglutaminase-1 (TGase-1) enzyme, which consists of 817 amino acid residues, resulting in a molecular weight of 90 kDa. In keratinocytes, the detectable full-length form of TG1 has been reported to be either 90 kDa or 106 kDa based on its running behavior in SDS-gels [34]; however, the current consensus is that the 106 kDa form likely represents a posttranslationally modified form of the 90 kDa core protein rather than a different size core protein [33,35]. The full-length TG1 is bound to the inner leaflet of the plasma membrane and requires proteolytic cleavage at two specific sites for activation in keratinocytes. The cleavage occurs at two sites; the residue 90 (Gly93) and residue 573 (Gly 573) generates three different-sized protein fragments, 10 kDa, 67 kDa and 33 kDa [34,36]. The 67 kDa fragment that contains the enzyme’s active site has approximately five times higher activity than the full-length form. A complex of the 67 kDa and 33 kDa fragments, which has been reported to be ten times more active, also exists. The 33 kDa fragment alone has no activity. The 67 kDa fragment begins at position 93, 15 residues before the predicted β-sandwich domain of TG1, while the 33 kDa fragment starts at the junction of the active site and β-barrel 1 domains. The 10 kDa N-terminal fragment, containing the membrane anchorage, remains inserted to the cell membrane after the fragmentation. The 67 kDa and 67/33 kDa forms contribute to 80–90% of the total soluble and cytosolic TG activity and 40–50% of the total cellular TG activity in differentiating keratinocytes [17,19,33]. These active forms in the cytosol can be recycled back to the membranes, facilitated by re-acylation of the 10 kDa fragment before degradation [34,37].

Early studies by Negi et al. (1981) first suggested that the activation and cleavage of TG1, ‘epidermal transglutaminase’ (as they referred to it), are mediated by lysosomal acid proteinases like cathepsin B and D. These enzymes are released and activated during the autolytic phases in the epidermal granular layer [38]. Research by Egbert et al. also indicated the involvement of cathepsin D in the epidermis, showing that levels of cornified envelope proteins and TG1 activity are severely diminished in the epidermis of Cathepsin D null mice where the 33 kDa TG1 protein fragment was undetectable [19]. This supports the hypothesis that Cathepsin D is crucial for generating the 33 kDa form of TG1. Additionally, calpain (a class of calcium-dependent intracellular cysteine proteases) has been also shown to regulate TG1 activity. Calpain may affect TG1 indirectly by modifying TG1 itself or its substrates [19]. It is not known if and how TG1 is activated in other cell types; however, Cathepsin D (or other aspartate proteases) may be involved at least in its function in osteoclasts (more in Section 13).

## 4. Identification of Substrates and Activity of TG1

As outlined above, TGs modify the structure, function, solubility and localization of their specific substrate proteins [1]. Identifying TG substrate proteins and their reaction products is essential for understanding the physiological impact of TGs and clarifying the specific roles of each enzyme. Each enzyme in the TG family appears to have their specific substrate proteins, as well as their specific glutamine residues within the substrates. However, it is still not understood how TGs select their substrates, or why only some glutamine residues are TG-reactive. There is no consensus sequence immediately around the TG-reactive glutamines, but it is possible that the recognition and binding ‘code’ is embedded into yet unknown sites within the 3D protein structure. The reactive glutamine-residues can be identified using various methods, including primary amine labeling of proteins followed by biochemical pulldowns and mass spectrometry, which has accelerated the identification of substrate proteins and labeled glutamine residues in vitro. Numerous substrates have been discovered this way in various tissues for TG enzymes; however, these tools have not been able to distinguish between substrates of different enzymes. A significant breakthrough in identifying TG-enzyme specific substrates was made by the Hitomi group, which screened an M13 phage-displayed random 12-mer peptide library to identify the preferred glutamine-containing sequences for each TG. These 12-amino-acid peptides serve as glutamine-donor substrates with specificity and high reactivity, allowing for the evaluation of individual TG enzyme activity. The most efficient substrate sequences screened from the random peptide library were F11 (FXIII-A), K5 (TG1), T26 (TG2), E51 (TG3), Y25 (TG6), and Z3 (TG7). A negative control to these peptides is obtained by replacing the glutamine residue (Q) with an asparagine residue (N) to prevent reactivity to TGs. These peptides are now widely used to evaluate individual TG activity in microtiter plate-, Western blot- and immunofluorescence-based assays, and for the visualization of in situ TG-specific activity [30,39,40,41,42]. Additionally, this system can be used as an efficient method to detect, purify and identify lysine-donor substrates in a TG-specific manner. Streptavidin-immobilized gel chromatography is employed to purify potential lysine-donor substrates from the total reaction products, followed by mass spectrometry for identification. TG1 substrates using the K5 peptide have been identified in cultured keratinocytes, kidney fibrosis and livers [42,43,44]. These advanced techniques enable researchers to gain deeper insights into the specific functions and regulatory mechanisms of TG1 and other transglutaminases in various biological contexts.

TG1 is expressed in several tissues, including the skin, dermal fibroblasts, lungs, kidneys, heart, and liver [2,45,46,47]. The use of fluorescent-labeled K5 peptides has been successful in detecting specific activities of TGs, including TG1, in frozen tissue sections. In these assays, a lysine-donor substrate within the tissue section covalently incorporates a glutamine-donor peptide, releasing a fluorescent signal that indicates the presence of active TGs [48]. Applying these fluorescent-labeled substrate K5 peptides to determine the mouse embryonic expression pattern of TG1 revealed its expression in epithelial tissues such as the skin, esophagus, tooth, and forestomach during embryonic development. Notably, apparent signals for both TG1 activity and protein expression have also been observed in hair follicles [48]. Due to the discoveries of TG1 activity in these tissues, the interest in the possible roles of TG1 beyond skin has been increasing in recent years. TG1 has been now associated with various disease states, including fibrosis, neurodegenerative diseases and certain types of cancers [45,49,50,51,52,53]. Below, we discuss the role of TG1 in skin and possible novel roles in other tissues and pathologies.

## 5. TG1 in Skin

TG1 was initially discovered and described in epidermal keratinocytes, where TG1 was shown to be produced around the time of 11–12 weeks of human fetal development [16,18,54]. The outermost layer of skin, the stratum corneum, acts as a crucial protective barrier, which prevents the entry of foreign substances and maintains fluid balance. This barrier is formed through a process called keratinization, where cells undergo distinct morphological and biochemical changes as they transit through various layers to create the cornified cell envelope, essential for proper skin barrier function [55]. The cornified envelope is formed by the crosslinking of proteins such as periplakin, envoplakin, loricrin and small proline-rich proteins (SPRRs) near the plasma membrane. By subsequent crosslinking of several structural proteins like involucrin, loricrin and keratin into the cornified envelope, TG1 stabilizes this 15-nm-thick structure that acts as a protective barrier between the body and its environment [18,36,55,56,57,58].

TG1 is highly expressed in several suprabasal layers of the epidermis, particularly the granular and upper spinous layers, where its activity is mainly required. However, its catalytic activity is primarily required in the uppermost granular layer during the initiation of keratinocyte cornification. To prevent premature protein crosslinking, TG1 activity must be tightly regulated. TG1 is initially synthesized as an inactive, soluble zymogen at low levels in proliferating keratinocytes, with its expression increasing progressively during keratinocyte differentiation. This process is regulated by tarazotene-induced protein 3 (TIG3), which controls terminal differentiation by activating TG1 [29,59]. TG1 undergoes proteolytic cleavage during differentiation, forming a highly active complex of 10/67/33-kDa fragments (described above). This activated TG1 facilitates the crosslinking of cornified envelope proteins and binds the terminal (omega) hydroxyl group of long-chain omega-hydroxyceramides to cornified envelope proteins. TG1 is inactivated upon terminal differentiation, likely because it integrates into the cornified envelope or is broken down by lysosomal proteases [60]. Moreover, *TGM1* gene expression in keratinocytes is regulated by a distal region of the *TGM1* gene promoter that contains AP-1 and Sp1 binding sites [61]. High calcium concentration is necessary for catalytic activity of TG1, which results in crosslinking of substrate proteins in the granular layer of the epidermis [62].

The distribution of the cell envelope precursor protein at the cell periphery depends on TG1 and cannot be replaced by other TG isozymes. To date, no cornified envelope defects have been linked to genes encoding other TGs [56]. In the absence of TG1, biochemical and amino acid composition changes occur in the skin that affect cornified envelope formation and composition. For instance, reduced levels of Ser and Gly indicate a decrease in the incorporation of proteins like loricrin, filaggrin and keratin into the cornified envelope. Conversely, elevated levels of Gln and Leu suggest a higher rate of crosslinking for involucrin in *Tgm1-/- mice*. These results highlight the importance of TG1 in crosslinking and formation of the cornified envelope [63,64]. Furthermore, by covalently crosslinking -OH ceramides with involucrin, TG1 may be required to maintain the function of the epidermal barrier [65].

Targeted deletion of TG1 in mice and inactivating mutations of human TG1 lead to aberrant cornified envelope formation and stratum corneum defects. *TGM1* mutations in humans result in lamellar ichthyosis characterized by scaly and thick skin. This deficiency impairs the formation of both lipid envelopes by forming ester bonds as well as protein envelopes, which are vital for healthy skin [26]. Different mutations, including missense, nonsense and splice site mutations, are located in exons 2, 5 and 11 of the TG1 enzyme, corresponding to the N-terminal and central catalytic domains. These mutations impact the catalytic core and disrupt TG1’s enzymatic activity, leading to ichthyosis [21]. Additionally, some mutations in the β-barrel domains disrupt inter-domain interactions, causing structural instability in TG1 [21,28]. In a cohort of kindreds with ichthyosis, several clinical characteristics associated with *TGM1* variants were identified [66]. Individuals with *TGM1* disease-associated variants were more likely to present with collodion membrane at birth and experienced a higher prevalence of hearing loss, eye problems, alopecia, and skin odor [66]. Moreover, due to the essential role of skin in synthesizing vitamin D, the children with congenital ichthyosis are at high risk of developing vitamin D deficiency and have indeed been reported to have lower vitamin D levels [67,68,69]. The vitamin D deficiency can lead to hyperparathyroidism and can result in poor bone mineralization in children and adolescents, leading to conditions such as rickets [67,68,69]. Additionally, a study on normal human keratinocytes has shown that active vitamin D3 promotes skin barrier formation by increasing TG1 expression and activity. This contributes to keratinocyte differentiation and enhances the protective function of the skin, though the exact mechanisms of this regulation remain unclear [70]. In addition to TG1, TG3 and TG5 are also expressed in human epidermis and contribute to cornification. Mutations of TG3 and TG5 cause only relatively mild epidermal phenotypes [71,72,73]. TG3 is expressed in the suprabasal layers of the stratified squamous epithelium, where it is involved in crosslinking of various structural proteins such as SPRs and loricrin within the cytoplasm [74]. These crosslinked proteins are then translocated to the cell periphery, where TG1 further stabilizes them by crosslinking them to the existing protein scaffold on the plasma membrane. This sequential and cooperative action between TG1 and TG3 is crucial for the complete and proper formation of the cornified envelope [75].

A pivotal study demonstrated that the activation of the TG1 gene is essential for facilitating skin injury repair during wound healing [76]. In this study, skin grafting was performed by excising dorsal skin from both control and *Tgm1-/-* neonatal mice, followed by transplantation onto athymic nude mice. Wounds were then made at the center of each graft using a biopsy punch. Histological experiments on harvested skin revealed substantial delays in wound healing in the *Tgm1-/-* mice [76]. The TG1 gene is activated in the early phases of wound healing, likely due to cytokines released from keratinocytes in response to cell damage or mechanical stimulation. Therefore, TG1 is critical not only for epidermis development and maturation but also for the progression of cutaneous regeneration [76].

Current treatments for ichthyosis are limited. The development of highly branched poly(β-amino ester) (HPAE) gene delivery vectors shows promise in delivering the *TGM1* plasmid, promoting efficient expression of TG1 protein in cutaneous cells, which is significant for treating inherited skin diseases like lamellar ichthyosis [77]. Additionally, tofacitinib, a broad-spectrum Janus kinase (JAK) inhibitor, has been suggested as a potential treatment for *TGM1*-associated autosomal recessive congenital ichthyosis (ARCI), characterized by elevated transepidermal water loss [78].

In tissue-specific autoimmune diseases, autoantibodies often target proteins that are crucial in the affected tissue. Identifying the connections between TG1 tissue localization and autoantibody reactivity in autoimmune bullous skin diseases has highlighted TG1 as a candidate autoantigen [79]. This is particularly relevant in severe blistering diseases such as paraneoplastic pemphigus [79].

Recent work by Alharbi et al. [80] has also suggested a role for TG1 in psoriasis, an autoimmune and inflammatory skin disease characterized by a thickened epidermis (acanthosis) due to amplified proliferation of keratinocytes, elongation of epidermal ridges, and retention of keratinocyte nuclei in the stratum corneum. In this study, TG1 activity, protein levels and mRNA levels were all elevated in psoriatic skin samples, suggesting these elevations as a compensatory response to the hyperproliferation and defective differentiation of keratinocytes [80]. In another recent study, the skin barrier disturbance in psoriasis was shown to stimulate a transient barrier reform response, marked by increased expression of various cornification-related proteins [81]. Additionally, in a third study, TG1 expression was observed in the lower epidermal layers of psoriatic skin, correlating with disrupted barrier function, and this expression can be regulated by Psoriasin (S100A7), a protein known to modulate differentiation markers [82].

Atopic dermatitis is a common chronic inflammatory skin disorder and often is considered as the first stage of allergic conditions like asthma and allergic rhinitis as individuals age. A key feature of atomic dermatitis is epidermal barrier dysfunction, which increases skin permeability to allergens, leading to inflammation and disease progression [83]. Several studies have reported elevated expression of TTG1 in the skin of atopic dermatitis patients sensitized to the skin-colonizing yeast Malassezia sympodialis (Mal s). This suggests that increased TG1 expression may serve as a compensatory mechanism in response to the compromised skin barrier [84,85].

## 6. TG1 in Vasculature

The vascular system maintains blood supply throughout the body, vascular integrity, as well as appropriate elasticity and vessel structures controlled by endothelial cells at the intima, which is in contact with blood. The maintenance and stability of this junction depends on the effective stabilization of cytoskeletal and extracellular matrix proteins, a process crucial for vessel remodeling and barrier maintenance [46,86]. TG1 has been shown to play a role in maintaining the endothelial barrier function, while TG2 activity is implicated in the inward remodeling of small arteries in response to low blood flow. Additionally, as atherosclerosis progresses, TG activity becomes increasingly important for a number of critical processes, including endothelial cell response, monocyte adhesion, vascular remodeling and arterial plaque stability [87]. TG1 and TG2 have been found in the aorta and vena cava, with their protein expression and activity reduced in the aorta of hypertensive rats [88]. Notably, TG1 is found in mouse myocardial endothelial cells, where it gradually accumulates at the junctional surface during monolayer maturation, with this monolayer acting as a cellular barrier that separates the blood compartment from surrounding tissues. TG1 enhances endothelial barrier stability against permeability-increasing stimuli, such as lowered [Ca^2+^]e and increased [Ca^2+^]i, by translocating to intercellular junctions [46,89,90].

Another study showed the expression of TG1 in smooth muscle cells, which play an important role in maintaining blood pressure via regulating the mechanical properties of blood vessels. This study also showed that the expression of TG1 is increased in response to mechanical stretching, which supports its involvement in vascular remodeling and regulation of blood pressure [91].

## 7. TG1 in Lung and Nasal Systems

The maintenance of the airways and epithelial barrier within the lung is vital for protection against inhaled pathogens and to maintain respiratory function [92,93]. Lung or alveolar injury results in a low oxygen condition (hypoxia), which hampers repair and promotes inflammation [94]. TG1 is distinctly expressed in human lung tissue and normal bronchial epithelium in vivo in mice [45,95]. Several studies have indicated that TG1 overexpression occurs in lung diseases, such as in non-small-cell lung cancer tumor cells, suggesting its involvement in the cell-to-cell attachment of tumor cells [45]. It is now well documented that hypoxia induces TG1 overexpression in the lungs. Promoter analysis to identify the region of the human *TGM1* gene promoter responsive to hypoxia showed a putative hypoxia-responsive element (HRE) within the range of −1424 to −1408 of the *TGM1* gene. The induction of *TGM1* gene activity under hypoxic conditions, mediated by HIF-1, depends on this HRE [94]. Moreover, TG1 is associated with the upregulation of airway epithelial junction proteins, including ZO-1, occludin, as well as β-catenin and E-cadherin, suggesting that hypoxia-induced TG1 may play a protective function in pulmonary diseases [45,94,96]. It was suggested that TG1 can be a useful therapeutic strategy for maintaining the integrity of the pulmonary epithelium during lung injury [89].

Conversely, the connection between the expression of *TGM1* isoforms in the airway epithelium and allergic conditions like asthma and chronic rhinosinusitis with nasal polyps (CRSwNP) has been well documented [97]. In CRSwNP, the presence of eosinophils, as the major inflammatory cells in nasal polyps, plays a role in the development of eosinophilic tissue within nasal polyps [98]. It has been shown that *TGM1* is expressed and colocalized with tissue eosinophils, involving the maintenance and development of nasal polyps through fibrin polymerization. TG1 is upregulated in the nasal polyps of patients suffering from CRSwNP, suggesting that TG1 could be a therapeutic target for treating CRSwNP [97].

In summary, TG1 exhibits both protective and detrimental effects in the lungs and airways, depending on the context and specific conditions. The exact role of TG1 in these tissues is still questionable and requires further research to elucidate these opposing roles and better understand the effect of TG1 on pulmonary health and disease.

## 8. TG1 in Hemostasis

Hemostasis, i.e., cessation of bleeding due to blood vessel injury, culminates in fibrin clot formation, which is stabilized by TG activity from Factor XIII-A. The crosslinking of the fibrin network and covalent addition of other anti-fibrinolytic constituents to the clot increase its resistance to fibrinolytic activity. Recently, TG1 was demonstrated to have a fibrin crosslinking ability similar to that of Factor XIII-A. In a study on chronic rhinosinusitis with nasal polyps (CRSwNP), TG1 was shown to contribute to the development of fibrin mesh and the excessive formation of polymer protein in nasal polyps, indicating its potential role in abnormal fibrin deposition in pathological conditions (see above paragraph) [97].

## 9. TG1 in Neurodegenerative Diseases

Alzheimer disease is the most prevalent neurodegenerative disease in individuals older than 60 years of age, characterized by extracellular plaques of amyloid-β (Aβ) peptides and intracellular tangles of hyper-phosphorylated tau [84]. Familial Alzheimer’s disease cases reveal mutations in the amyloid precursor protein (APP) and presenilins (PSEN1 and PSEN2) involved in Aβ processing [99]. An aberrant transglutaminase (TG) enzymatic activity in Alzheimer’s disease, as well as other neurodegenerative disorders such as Parkinson’s disease and Huntington’s disease, has been demonstrated, but mostly linked to TG2 [100,101]. TG activity was demonstrated to primarily target Amyloid-β precursor protein (APP) and α-synuclein, leading to abnormal protein crosslinking and aggregation by altering their conformational and structural properties, which contributes to the pathogenesis of neurodegenerative diseases [102]. In Alzheimer’s disease, a significant increase in ε(γ-glutamyl)lysine crosslinks has been observed in tissue samples from the cortex and cerebellum of Alzheimer’s disease patients compared to controls [103,104]. TG1 is expressed in the brain and is transcriptionally upregulated in the hippocampus of Alzheimer’s disease mice models, such as in 3xTg-alzheimer’s disease mice [52,105], causing toxicity by reducing neuronal viability and altering the membrane excitability in an in vitro model of AD. This suggests a link to Alzheimer’s disease progression. The upregulation of TG1 is mediated by AP1-responsive elements at the *TGM1* promoter. Moreover, silencing TG1 in cultured primary neurons from an in vitro model of Alzheimer’s disease was demonstrated to prevent Aβ-mediated neuronal death [52,101,106].

Beyond Alzheimer’s disease, TG1 was also shown to be involved in hyperphosphorylated tau protein inclusions in tauopathies such as Progressive Supranuclear Palsy (PSP) and Frontotemporal Dementia linked to chromosome 17 (FTDP-17). In early stages of fibrillary tau formation in neurons, TG1, along with TG1 activator TIG3, catalyzes the crosslinking of tau proteins, leading to the formation of stable tau aggregates and increasing neurotoxicity and leading to neuronal death in these tauopathies [107].

TG1 was also shown to be present in corpora amylacea, which are age- and neurodegeneration-related spherical bodies involved in the sequestration of hazardous products of cellular metabolism [108]. In the brain, TG1-catalyzed crosslinking and polymerization of cytoskeletal and cytoskeleton-associated proteins are suggested to contribute to the formation of corpora amylacea [109].

## 10. TG1 in Cancer

TG1 has been implicated in the progression of various cancers, including gastric cancer, a prevalent malignancy that ranks among the leading causes of cancer mortality globally [53]. Research has shown that the loss of *TGM1* inhibits gastric cancer cell proliferation in human cancer cells, where it increases apoptosis by arresting cells in the G0/G1 phase and altering the expression levels of Bcl-2 and Bax [53]. Furthermore, TG1 deficiency enhances the sensitivity of gastric cancer cells to chemotherapeutic drugs and reduces stemness [53]. There is also evidence suggesting that TG1 may regulate gastric cancer through the Wnt/β-catenin signaling pathway, as the loss of *TGM1* significantly inhibits Wnt/β-catenin signaling, which is linked to enhanced cell proliferation and decreased cell death [53,110].

Beyond gastric cancer, TG1 has been linked to cancer via its upregulation in various tumors, including ovarian cancers, where it may contribute to metastasis and tumor progression through catalyzing protein crosslinking [111]. Additionally, TG1 expression is elevated in cervical squamous cell carcinoma and endocervical adenocarcinoma (CESC), as well as in thyroid carcinoma (THCA) [112]. Knockdown of *TGM1* in cancer cells has been shown to enhance apoptosis, indicating its involvement in tumor progression [53]. Notably, high expression levels of *TGM1* correlate with a poor prognosis in several cancers, including skin cutaneous melanoma [113], whereas in some cases, such as in Glioma (GBMLGG), TG1 appears to act as a protective factor [113]. This variability in TG1’s role in cancer was suggested to be influenced by epigenetic regulation and tumor heterogeneity [53,114].

Additionally, *TGM1* is linked to immune responses within the tumor microenvironment, which potentially impacts the effectiveness of immunotherapy [113]. A pan-cancer analysis using data from The Cancer Genome Atlas (TCGA) revealed a significant correlation between *TGM1* expression and the infiltration of CD4+ T cells, CD8+ T cells, neutrophils and dendritic cells in Bladder Urothelial Carcinoma [112,113] and Breast Invasive Carcinoma [113]. As a result, *TGM1* is being investigated as both a prognostic marker and a therapeutic target for cancer treatment [113].

A recent study demonstrated that TG1, jointly with TG3, mediates posttranslational transamidation of key proteins such as Exo70, a crucial component of the exocyst complex and essential for tumor cell migration and invasion [115]. This crosslinking modification enhances the interaction of Exo70 with other exocyst subunits, resulting in increased matrix metalloproteinase secretion, invadopodia formation and extracellular matrix degradation, which are vital for cancer metastasis. Interestingly, this study showed that the Exo70 modification is suppressed by liver kinase B1 (LKB1), a tumor suppressor often inactivated in various cancers, which phosphorylates TG1 and TG3 [115]. This phosphorylation by LKB1 induces a conformational change in TG1 and TG3 active sites, which inhibits their interaction with Exo70 and thereby prevents its transamidation [115]. This study is the first to demonstrate that TG activity can be inhibited by phosphorylation.

These findings underscore the importance of considering TG1′s function within the specific target tissue when evaluating its role in cancer, as the tumor immunological environment and tissue-specific function have a major influence on its impact on carcinogenesis.

## 11. TG1 in Fibrosis

The hallmarks of chronic kidney disease (CKD), which include glomerulonephritis and diabetic nephropathy, are glomerulosclerosis and tubulointerstitial fibrosis. These conditions arise from progressive remodeling processes, such as excessive extracellular matrix protein accumulation [116]. TG1 has been also implicated in the induction of renal fibrosis via a mechanism that involves extracellular matrix stabilization and the activation of TGF-β1 [43]. Studies have shown that TG1 is highly expressed and activated in a renal tubular epithelial cell line under oxidative stress [47,117]. TG1 was demonstrated to contribute to fibrotic kidney diseases, with its activity increasing in the renal tubular epithelium at the early fibrotic stage and in the interstitial areas (the space between blood vessels and renal tubules) at the late fibrotic stages [117]. This increase in TG1 activity was confirmed through its colocalization with the renal epithelial marker E-cadherin [117]. Detection of possible TG1 substrates in fibrosis promotion via mass spectrometry identified several substrates involved in renal disease and fibrosis, such as Fibrinogen β chain, Keratin 5, Protein S100-A9, Serotransferrin, Tubulin α-1C and Tubulin β-5. Additionally, when categorized by protein class, the majority of TG1-specific substrates were identified as cytoskeletal proteins and actin family cytoskeletal proteins [43]. These findings suggest that TG1 plays a role in the progression of kidney fibrosis by modifying proteins that contribute to extracellular matrix stabilization and the progression of fibrosis [43].

Additionally, the role of TG1 in cardiac fibrosis, a hallmark of most forms of cardiovascular disease characterized by pathological remodeling and excessive accumulation of extracellular matrix proteins within the myocardium, is suggested by its concomitant expression with TG2 and its correlation with markers of fibrosis progression. While TG2 is well-studied in fibrotic conditions, TG1’s transcript levels also correlate with collagen content, fibronectin and connexin 43, indicating its influence on extracellular matrix remodeling and fibrosis progression [118,119]. This is further supported by findings from experiments in neonatal rat fibroblasts and cardiomyocytes, where TG1 knockdown led to reduced insoluble collagen levels, decreased collagen crosslinking and altered profibrotic marker levels, indicating TG1′s functional impact on extracellular matrix dynamics. This indicates that TG1, like TG2, may influence extracellular matrix remodeling and fibrosis progression in cardiovascular diseases [88].

In a study on liver fibrosis, TG1 activity was found to be significantly elevated in the parenchymal cells of the liver following bile duct ligation (BDL), with a distinct localization with collagen in the fibrotic liver tissue. This enhanced TG1 activity was widespread across the fibrotic liver sections, indicating its potential role in the fibrotic process [120].

## 12. TG1 in Innate Immunity: Neutrophils

Neutrophils, which serve as a first line of defense, become activated by various inflammatory stimuli and exert their protective function via several mechanisms, including forming Neutrophil Extracellular Traps (NETs), a process which is referred to as NETosis. During NETosis, neutrophils release decondensed DNA and other constituents in the form of a web-like structure, which enables them to trap and eliminate pathogens. During this process, endogenous polyamines are covalently incorporated into both cellular and NET-specific proteins, a process dependent on myeloperoxidase activity [121,122]. TG1 was demonstrated to be present in neutrophils and was suggested to play a role in NETosis via stabilization of a broad set of NET proteins and via adding polyamines to the NET proteins via a transamidation reaction. TG1 is also expressed in circulating neutrophils, further supporting its involvement in neutrophil function [122].

## 13. TG1 and Regulation of Bone Mass

Bone remodeling is a dynamic process crucial for maintaining bone quality and strength throughout life. Bone remodeling involves the continuous degradation of old bone by bone-resorbing cells, osteoclasts, and its replacement with new bone by bone-forming cells, osteoblasts. Cellular defects in bone remodeling and imbalanced coupling between the bone cells result in osteoporosis, a condition characterized by loss of bone mass and increased risk of fractures. Recent studies have highlighted a novel role of TG1 in bone biology, particularly in the regulation of osteoclastogenesis, i.e., the development of bone resorbing cells, osteoclasts from monocyte-macrophages. TG1 was demonstrated to be present and active in bone marrow monocytes, macrophages and osteoclasts, and it is expressed throughout osteoclastogenesis [123,124]. Its expression was demonstrated to have a distinct pattern during monocyte-colony stimulating factor (M-CSF)-induced macrophage differentiation and M-CSF/receptor activator of nuclear factor κB ligand (RANKL)-induced osteoclast differentiation. TG1 expression significantly increases with M-CSF treatment in macrophages and with M-CSF and RANKL treatment in osteoclasts, though the upregulation by M-CSF alone is higher than with M-CSF/RANKL [124,125] [98,100]. TG1 colocalized to podosomes (actin-containing structures involved in adhesion and cell migration) in osteoclasts [126]. The role of TG2 and Factor XIII-A in bone biology was demonstrated in a knockout model lacking both enzymes in *Tgm2-/-/F13a1-/-* mice [123]. These mice had dramatic loss of bone mass due to the increased osteoclast formation potential of monocytes in vitro, which were demonstrated to express the TG1 enzyme. The increased potential was completely reversed by TG inhibition with NC9, suggesting that TG1 was the active enzyme driving osteoclastogenesis. Additionally, decreased bone mass was also observed in *Tgm2-/-* mice in vivo, which was caused by increased osteoclastogenesis [127]. The TG2-deficient monocytes and bone marrow macrophages showed significant upregulation of TG1 mRNA expression and activity (as measured with K5 peptide). Moreover, inhibition of osteoclastogenesis of *Tgm2*-/- monocytes by NC9 also blocked the osteoclastogenic program [128]. Furthermore, it was also demonstrated that wild type and *Tgm2-/-/F13a1-/-* bone marrow mesenchymal stem cells (BMMSCs) express TG1. The mesenchymal stem cells showed increased potential for adipogenesis as well as increased RANKL expression. It is not known if TG1 is involved in any of these observed alterations [123].

## 14. TG1 in the Visual System

The involvement of TG1 in the development of human eye and function was also studied recently. Proliferative vitreoretinopathy (PVR) is characterized by the formation of scar-like fibrocellular membranes on the retinal surface, in the vitreous, and in the subretinal space, which causes detachment of the retina and loss of vision [129]. TG1 was shown to be present in the membranes of PVR and to contribute to the membrane development via increasing cell adhesion and the formation of a fibrotic extracellular matrix. The dedifferentiation of retinal pigment epithelial (RPE) cells to fibroblast-like cells seems to be a main pathological event in PVR disease. It was shown that the dedifferentiation of RPE and an increase in TGF-β2 levels, a pluripotent cytokine regulating several biological activities involved in the pathogenesis of PVR, can alter TG1 expression in the RPE [130].

**Figure 3:**
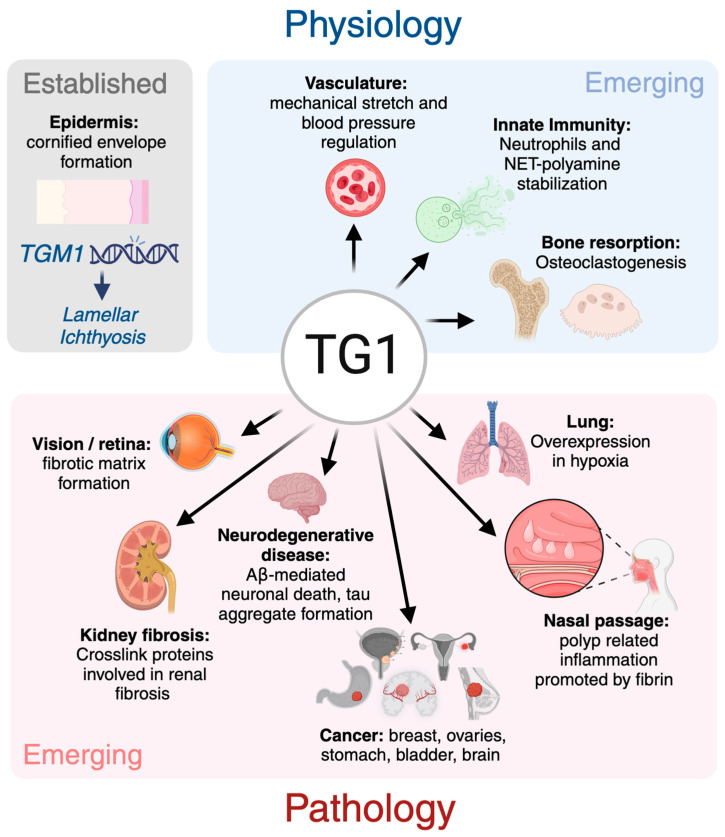
Summary and overview of the established and emerging roles of TG1 across various tissues. Beyond its established function as stabilizer of the skin barrier, novel roles are emerging in both normal physiological settings and in pathologies. Created in BioRender. Kaartinen, M. (2024) BioRender.com/i64w351.

## 15. Future Perspectives

**Figure 4:**
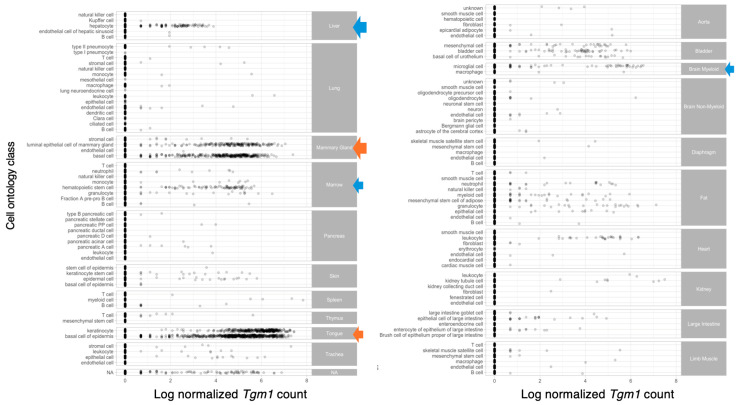
Whole-body expression pattern of mouse *Tgm1* mRNA identified by data mining of the scRNA sequencing atlas Tabula Muris project [118]. The analysis reveals strong expression (orange arrows) in tongue epidermis and keratinocytes as expected, and in luminal epithelial cells and basal cells in the mammary gland. Moderate expression (blue arrows) is also seen in hepatocytes in the liver, hematopoietic stem cells in the bone marrow and in brain microglial cells. Each dot represents a sequenced cell.

The novel roles of TG1 are emerging in several physiological and pathological processes (Figure 3). The expression patterns of TG1 in the skin, lungs, kidneys, cardiovascular system and differentiating cells, such as in bone, further emphasize that TG1 has physiological significance in normal tissue homeostasis. Our new data mining from the Tabula Muris single cell RNA sequence Atlas (Figure 4) indicates that TG1 is also normally expressed in hepatocytes, mammary glands, bone marrow and pancreases in mice, which suggests more physiological roles, meriting further explorations [131]. The roles of TG1 are emerging also in several pathologies summarized in Figure 3, suggesting it as a therapeutic target. 

The development of a *Tgm1*flx/flx mouse model will allow for development of tissue-specific conditional knockout models to explore these novel functions in vivo. TG1 substrate identification through advanced techniques such as mass spectrometry and peptide library screening will continue to enhance our understanding mechanisms of action of TG1 in these various biological contexts. Remaining questions also include understanding the need for TG1 activation in systems outside skin and in the extracellular matrix. Also, the novel and recent discovery by Hou et al. on the regulation of TG1 (and TG3) transamidase activity via kinase-mediated phosphorylation opens a concept of a novel modulatory system that may also regulate activity of other TGs. Specific inhibitor development can result in tissue-targeted therapies and ultimately boost outcomes for patients with TG1-related conditions as well as deepen our understanding of the complex roles of TG1 in human disease processes.

## Data Availability

Not applicable.
